# Human resources for eye care: changing the way we think

**Published:** 2009-03

**Authors:** Usha Raman

**Affiliations:** Associate Director and Head, Communications, LV Prasad Eye Institute, Banjara Hills, Hyderabad 500 034, India.

**8^th^ General Assembly of IAPB**

**Course 10:** Productivity of eye care workers

**Speakers:** Paul Courtright, Daniel Etya'ale, Hannah Faal, Ingrid Mason, Van Lansingh, Noel Chua

**Figure:**
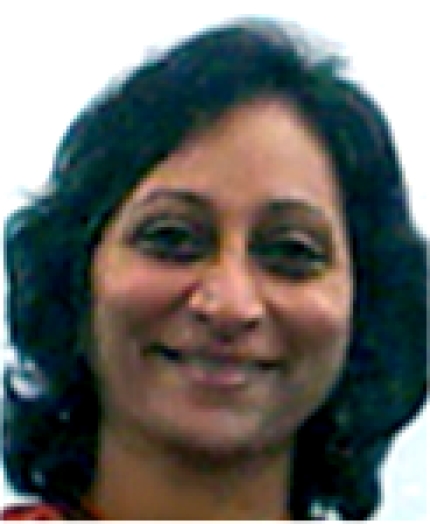


The question of human resources is central to the success of VISION 2020 and of any health programme.

The VISION 2020 global initiative document clearly spelt out what personnel was required and, more recently, the World Health Organization document on human resources for health care made recommendations on the type and number of people needed in order to meet all our objectives in global health. In spite of this, practically none of the national eye care policies articulated so far have a clear recommendation on human resources.

## Numbers and distribution

Traditionally, much of the discussion on human resources for eye care has focused on numbers and distribution. Questions of how many and what kind of people we need, what sort of training they need to be given, and where we need them, dominate the conversation.

There is still a great need for eye health personnel in most countries. The reasons for this ‘human resource crunch’ vary worldwide, from a dearth of people suitable for training to high levels of emigration of trained personnel - such as occurs in the Philippines, where the export of human resources, particularly nurses, has led to the closure of hospitals and services.

However, although the issue of numbers and distribution of personnel is still an important one, our thinking about human resources is still very much ‘from the top down’. As one speaker noted, there is an urgent need to shift our focus towards those who need care.

## A shift of focus towards the needs of communities

Daniel Etya'ale remarked that we need to think not of eye care workers, but of ‘personnel needed for eye care’. Noting that the former label was too restrictive, he proposed the latter term, to move the focus to those who need care rather than those who provide it. This stems from a larger shift in focus from inputs to outcomes. The training and deployment of health workers then becomes oriented to community need.

Hannah Faal also emphasised the need to step out of the ‘eye box’ and train professionals who can be integrated into other systems. This is particularly important at the primary level of service delivery, where integrated health care is key to achieving progress.

**Figure:**
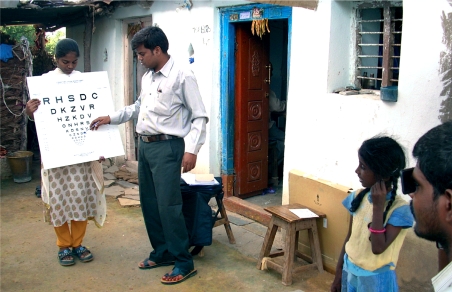
Vision testing by a community eye care team. INDIA

International Centre for the Advancement of Rural Eye Care

## New cadres

Early on in the VISION 2020 initiative, it was recognised that we need to develop new cadres of professionals who will work with, and within, communities to stem the rising tide of avoidable blindness.

However, eye care service delivery at all levels still relies too heavily on ophthalmologists, though the degree of this dependence on medical specialists varies across the globe. It is becoming increasingly clear that, if we wait for enough ophthalmologists to be trained and appointed to take care of all forms of vision impairment, the goals of VISION 2020 will not be met.

Midlevel ophthalmic personnel, vision technicians, ophthalmic nurses, nursing aides, instrument technicians, as well as ‘hybrid’ professionals who can perform a variety of functions, are all essential if we are to meet the human resource need for eye care.

The experience in Latin America suggests that productivity is limited when dependent on individual ophthalmologists. Eye care teams with flexible job descriptions and common goals work better in most situations, as certain categories of professionals (such as optometrists) are entirely absent in this region.

Van Lansingh explained that often team members have no job description, but build a combined commitment to outcomes and practise effective communication. These are factors which drive high performance.

## Productivity of eye care workers: maintaining enthusiasm

Even where the numbers of personnel are adequate and there is some degree of focused training, productivity remains a challenge. Ingrid Mason spoke of the need to examine, in each specific context, the possible causes of low productivity and its relationship with the quality of care.

Experience has shown that while enthusiasm, and consequently, productivity, is high at the beginning of a new entrant's placement, this fags very soon thereafter. Problems such as community resistance, isolation from professional and academic bodies, and difficulties in dealing with technology and equipment, are often responsible for the fall in enthusiasm.

It is necessary to re-examine the content and delivery of training programmes, as well as the practicality of criteria for evaluation. Much of the demand for training and the frameworks for evaluation must be dictated by the experience and expectations of community eye health workers.

If community health workers are to deal with eye care and perform basic vision screening, then they should be equipped with the skills and the technology to do this. They may also need to be given the technical wherewithal to manage and maintain equipment, as well as the advocacy and communication skills to create and exploit links with other sectors, when and where required.

Talk of productivity, therefore, can happen only if there is a clear understanding of what needs to be done and by whom, and it must be grounded in the realities of the community.

